# The complex interplay between psychosocial and biological factors in pregorexia nervosa — a rapid review

**DOI:** 10.3389/fpsyg.2023.1168696

**Published:** 2023-06-19

**Authors:** Octavian Vasiliu

**Affiliations:** Department of Psychiatry, Dr. Carol Davila University Emergency Central Military Hospital, Bucharest, Romania

**Keywords:** pregorexia nervosa, anorexia nervosa, eating disorders, pregnancy, orthorexia nervosa, self-image distortions, ideal body image

## Abstract

The importance of detecting eating disorders (EDs) during pregnancy cannot be overemphasized, because of the major negative effects this pathology has on both maternal and fetal health. Based on a rapid review including primary and secondary reports, PN may still be considered an elusive diagnosis entity, that partially overlaps with other EDs, either well-defined, like anorexia nervosa, or still in search of their own diagnosis criteria, like orthorexia nervosa. Neurochemical and hormonal factors, psychological and social mechanisms, along with lifestyle changes create a very complex framework for clinicians interested in defining the typical features of pregorexia nervosa (PN). The personal history of EDs is considered one of the most important risk factors for PN. The core diagnostic criteria for this entity are, so far, lack of gaining weight during pregnancy, an excessive focus on counting calories and/or intense physical exercising with a secondary decrease of interest in the fetus’s health, lack of acceptance of the change in body shape during pregnancy, and pathological attention for own body image. Regarding the treatment of PN, nutritional and psychosocial interventions are recommended but no specific therapeutic strategies for this disorder have been detected in the literature. Psychotherapy is considered the main intervention for pregnant women with associated EDs and mood disorders, as the pharmacological agents could have teratogenic effects or insufficient data to support their safety in this population. In conclusion, taking into consideration the methodological limitations of a rapid review, data supporting the existence of PN were found, mainly regarding tentative diagnostic criteria, risk factors, and pathophysiological aspects. These data, corroborated with the importance of preserving optimal mental health in a vulnerable population, e.g., pregnant women, justify the need for further research focused on finding specific diagnostic criteria and targeted therapeutic approaches.

## 1. Introduction

Pregnancy is associated with multiple changes in self-image, social roles, professional schedule, and lifestyle activities but also in the physiological parameters; therefore this period can be conceived as a complex bio-psycho-social challenge for women, which involves the activation of stress coping mechanisms and other various mental resilience factors but also the risk of revealing biological or psychological vulnerabilities. Weight gain during pregnancy may trigger adaptive responses ranging from mild discomfort and a tendency to control this phenomenon using adequate strategies under appropriate medical supervision, to the onset of psychiatric disorders or syndromes, e.g., adjustment disorders with depressive or anxiety symptoms, dysmorphophobia, and eating disorders (EDs; Martínez-Olcina et al., 2020). The co-occurrence of anxiety/mood symptoms/disorders and EDs in this population is not infrequently reported, and their common ground could be represented by excessive concerns about weight gain (Martínez-Olcina et al., 2020).

The estimated prevalence of EDs in pregnant women was estimated to be between 5.1 and 7.5% (Easter et al., 2013; Martínez-Olcina et al., 2020). However, the epidemiological data varies largely, depending on the methodology used, i.e., descriptive self-report vs. structured and clinician-evaluated status, number of previous deliveries, and various demographic variables. For example, in a retrospective study focused on the exploration of the frequency of self-reported EDs and body changes in women who had been delivered three to seven months earlier (N = 454 respondents), a history of ED was admitted by 11.5% of the participants, and younger women were the most affected by this pathology (Larsson and Anderson-Ellström, 2003). Interestingly, no difference was reported in this study in the feelings related to the transformation of the body shape, and almost all women described these feelings as positive (Larsson and Anderson-Ellström, 2003). Therefore, these data suggest a positive effect of pregnancy on body weight changes, i.e., that women with a history of ED and those without such a history presented a similar favorable perception of their weight gain. Still, pregnancy is considered a factor that may complicate the evolution of an ED because changes in the body shape can trigger worries related to increased weight gain (Ward, 2008). As a consequence, caution is needed while monitoring pregnant women with an ED history because, while in certain cases this phase associates a reduction of body weight-focused worries, in other cases an increase of the same concerns may be observed. The exploration of specific vulnerability psychological factors, the psychosocial context of the current pregnancy, and a detailed history of the ED may be helpful in the early detection of favorable vs. unfavorable perceptions of body changes during this period.

The relationship between EDs and pregnancy is a very complex one, being mediated by multiple variables, e.g., prior distortions of the self-image, fertility problems, pre-existing and new-onset anxiety and depression, etc. (Ward, 2008). For example, women with anorexia nervosa have more difficulties conceiving, and there may be a delay between regaining normal weight and the return of menstruation and fertility; unplanned pregnancies may occur more frequently when periods are irregular and contraception methods are not routinely used; in patients with ED, the weight gain accompanying the pregnancy may worsen the symptoms (Rocco et al., 2005; Morgan et al., 2006; Micali et al., 2007; Ward, 2008). The inverse phenomenon (i.e., the improvement of the ED symptoms) may also appear due to the worries of pregnant women about the health of their unborn babies, which shift, at least partially, the focus of attention from their own body weight or shape to the development of the fetus. Although women with recent EDs continued to have anxiety about weight gain, dieting, using laxatives, excessive exercise, or self-induced vomiting during pregnancy, the severity of these symptoms was lower than before pregnancy (Rocco et al., 2005; Morgan et al., 2006; Micali et al., 2007; Ward, 2008).

EDs during pregnancy have been reported more frequently in young women with a history of ED and/or sexual abuse, who experienced significant life stressors or psychological traumas (Czech-Szczapa et al., 2015; Tuncer et al., 2020). According to a questionnaire-based study (*N* = 199 mothers of newborns hospitalized in a neonatal intensive care unit), scores higher than 20 on the Eating Attitudes Test-26 item-version (EAT-26) were also associated with smoking more often during the last period of pregnancy (Czech-Szczapa et al., 2015). EAT-26 is a commonly administered psychometric instrument to assess the risk of EDs, and its psychometric properties were found valid in clinical and non-clinical populations, and across cultural settings (Papini et al., 2022; McLean and Kulkarni, 2023). The factorial structure of EAT-26 was revised repeatedly, and models including one to six factors have been described (McLean and Kulkarni, 2023). Fewer women with adequate pre-gestation body mass index (BMI) were identified in the study group vs. the control group (N = 127 mothers of healthy newborns who “roomed in” with their mothers) and gained less weight during pregnancy (Czech-Szczapa et al., 2015).

EDs detected in pregnant women may lead to a significant risk for negative effects in mothers (e.g., maternal hypertension, anemia, postpartum depression) and children (e.g., low birth weight, premature births, spontaneous abortion, microcephaly, higher susceptibility to diseases later in life; Micali et al., 2009; Bakker et al., 2010; Watson et al., 2017; Martínez-Olcina et al., 2020; Tuncer et al., 2020; Janas-Kozik et al., 2021). More specifically, inadequate weight gain during pregnancy has been correlated with various maternal (e.g., cesarean section, miscarriage, diabetes, or abdominal adiposity) and fetal risks (e.g., fetal malformations, or abnormal fetal growth; Diemert et al., 2016). A balanced diet during pregnancy influences maternal body weight and health risks in the short-and long-term in the mother, as well as in the child (Diemert et al., 2016). EDs prior to or concomitant to the pregnancy, but also present or past depression were associated with higher anxiety levels and more severe depression perinatally (Micali et al., 2011). At 8 months post-partum, the presence of pregnancy ED symptoms and/or past depression was reported in women with the highest risk for probable depressive and anxiety disorder after childbirth (Micali et al., 2011).

For clinicians, it is, however, challenging to diagnose EDs in pregnant women based solely on routine examination, because manifestations of eating behavior pathology and physiological changes associated with pregnancy may overlap (Bannatyne et al., 2018). For example, changes in dietary preferences and eating patterns, food aversions or cravings, and decreased or increased appetite are reported by most women during pregnancy, but also by patients with EDs (Tiller and Treasure, 1998; Bannatyne et al., 2018). Care should be taken to not overpathologise adaptive responses to pregnancy, and differentiating healthy changes in lifestyle triggered by the need to control excessive weight gain from restrictive, rigid, and not-medically validated strategies for weight control is imperative. Therefore, collecting a detailed anamnesis, with a focus on specific eating habits and body image distortions, especially where symptoms of EDs may be suspected, is needed in this population. Also, the clinician is encouraged to compare more discrete symptoms with existing clinical guidelines, to evaluate possible dysfunctions in daily life that may be caused by such symptoms, and to determine the level of the patient’s awareness of her health problems based on behavioral cues (Bannatyne et al., 2018).

“Pregorexia” or “pregorexia nervosa” (PN) is a term coined by mainstream media in the summer of 2008, with the purpose of describing pregnant women who reduce their caloric intake and involve themselves in excessive physical exercising as a way to control pregnancy-related weight gain (CBS News, 2008; Mathieu, 2009; Fox News, 2015). A high level of worry related to the risk of becoming overweight and intense preoccupations with behaviors destined to mitigate this trend toward weight or body shape modifications related to a healthy pregnancy is considered central to this diagnosis (Mathieu, 2009). The social models advertised by mass media have been suggested as potential contributors to the onset of pregorexia, in the sense that achieving a „perfect” pregnant body is desirable and worth excessive working out or dieting (CBS News, 2008; Mathieu, 2009). Of course, the accent in pregorexia is placed on the disproportionate dieting or strenuous physical effort and does not include the normal, healthy adaptative strategies to the pregnancy period, i.e., using dietary supplements, exercising regularly, and avoiding harmful behaviors like smoking or alcohol use. This diagnosis is not formally recognized by the 5th edition of the Diagnostic and Statistical Manual of Mental Disorders (DSM-5) or by the 11th edition of the International Classification of Diseases [ICD-11; American Psychiatric Association (APA), 2013; World Health Organisation, 2023]. PN could still be classified as an „unspecified feeding or eating disorder” in the terminology of DSM-5 or as a „feeding or eating disorder, unspecified” in the framework of ICD-11 [American Psychiatric Association (APA), 2013; World Health Organisation, 2023].

Monitoring of the BMI in pregnant women is an important part of the overall periodic evaluations scheduled during this period. In patients with a history of EDs or with risk factors for such a disorder, the importance of BMI monitoring is even higher. According to the Institute of Medicine (IOM) weight gain recommendations for pregnancy (2009), in underweight women (BMI < 18.5 kg/m^2^) the admissible range of weight gain is 28–40 lb., with a mean rate of increase in the second and third trimester of 1–1.3 lb./wk.; in normal-weight women (BMI = 25–29.9 kg/m^2^) the usual range of total weight is 25–35 lb., with a mean rate of increase in the 2nd and 3rd trimester of 0.8–1 lb./week; in overweight women (BMI = 25–29.9 kg/m^2^) the recommended range of total weight is 15–25 lb., with a mean of 0.5–0.7 lb./week in the last two trimesters; in obese women (IMC > 30 kg/m^2^) the recommended range of total weight is 11–20 lb., with a mean of 0.4–0.6 lb./week (American College of Obstetricians and Gynecologists, 2013). The detection of women with signs of PN could be significantly facilitated by applying regular determination of the BMI during pregnancy, followed by an interview focused on dieting and exercise practices.

Lifestyle changes during pregnancy are quite diverse and may vary from (1) eliminating unhealthy habits to (2) orthorexia nervosa-feeding patterns or (3) food-specific cravings (Blau et al., 2020; Gerontidis et al., 2022). All these changes may be interpreted as coping strategies focused on decreasing pregnancy stress (Blau et al., 2020; Gerontidis et al., 2022). Therefore, the onset or exacerbation of EDs during pregnancy can be the result of activating dysfunctional coping mechanisms, which are intended to reduce the stressful consequences of physical, psychological, and social adjustments specific to this phase (Tiller and Treasure, 1998).

Also, maternal dietary patterns during pregnancy have been explored in relation to early childhood growth. A pattern defined by higher intake of fast food (i.e., fried chicken, fried fish, fruit juices, mayonnaise, and sugar-sweetened beverages) was associated with a rising-high BMI trajectory in children and/or the presence of obesity/overweight at age four (Hu et al., 2020). The processed food pattern (i.e., dairy, salad dressing, processed meat, and cold breakfast cereal) during pregnancy was not associated with early childhood growth and increased risk for obesity in offspring (Hu et al., 2020). In a cross-sectional study (N = 157 pregnant women) over 60% of the participants reported improvements in their diet since conception, but pica tendencies were also observed in isolated cases (e.g., consuming ice and snow) (Gerontidis et al., 2022). Orthorexic tendencies were present in this sample, especially in women who were pregnant for the first time (Gerontidis et al., 2022). In another study, food cravings were described by 68 pregnant women in their second trimester as cognitively demanding circumstances that could be differentiated from hunger (Blau et al., 2020). Emotional predisposing factors (both positive and negative emotional states) and reactions to environmental stimuli (selective appetite as a way to cope with psychological distress) have been described in relation to food cravings (Blau et al., 2020). The phenomenon of „food addiction” has not yet been explored systematically in pregnant women, but cravings for highly palatable foods are frequently reported in this population and may contribute to the onset or maintenance of gestational overweight or obesity (Haddad-Tóvolli et al., 2022). The exploration of this recently-cornered diagnosis in pregnant women would be of theoretical and practical interest because restrictive EDs and food addiction could share similar pathophysiological mechanisms. For example, several arguments have been substantiated about the addictive nature of anorexia nervosa: food restriction increases the reinforcing effects of drugs of abuse; there is a high rate of comorbidity between anorexia nervosa and substance abuse; both anorexia nervosa and substance use disorders present early onset during adolescence, and both begin with a conscious decision to engage in a certain behavior, i.e., use of a drug or starting a diet (Carr, 2002; Barbarich-Marsteller et al., 2011). Dopaminergic mesolimbic circuitry activation has been reported in mice with gestational food craving-like episodes (Haddad-Tóvolli et al., 2022). Pregnancy has been assumed to engage the modulation of dopaminergic neurotransmission in the nucleus accumbens, which directly influences food cravings via dopaminergic D2 receptors (Haddad-Tóvolli et al., 2022). Studies on food addiction in the general population highlight the relationship between salient foods that stimulate brain reward motivation and stress circuits, influencing eating behaviors, i.e., promoting food craving and excessive food intake (Sinha, 2018; Vasiliu, 2022a). High levels of stress may act on prefrontal cortical self-control processes regulating emotional, motivational, and visceral homeostatic mechanisms of eating and obesity, while the neurobehavioral changes can further support food craving and weight gain, in a feed-forward manner (Sinha, 2018).

The challenge of self-isolation during the COVID-19 pandemic has changed lifestyle and diet patterns in the general population for a significant period of time, and gradual re-adjustment is necessary (Vasiliu et al., 2021; Alimoradi et al., 2022). The risk of developing dysfunctional coping strategies is also not at all negligible, with a potential increase in behavioral and substance addictions being signaled in the literature (Alimoradi et al., 2022; Vasiliu, 2022b). Therefore, the importance of EDs in the post-COVID-19 pandemic period is increasing, if specific factors associated with that period are taken into consideration (e.g., worries about self-health, limited access to certain foods, decrease in social networking, etc.) and the need to re-adjust to normal life conditions can differently affect various populations. Pregnancy represents a vulnerable period that may trigger the activation of previously acquired dysfunctional feeding patterns and exploring such patterns learned during the self-isolation pandemic period can be useful for clinicians. PN can be detected by screening in pregnant women for recently implemented lifestyle changes, like severe eating restrictions, complete elimination of hypercaloric foods, and increased attention to eating consequences on own body; low propensity for food craving could be intuitively detected in this population, due to a more rigid approach to dieting.

Based on the need to find the characteristics of PN and to differentiate it from other EDs, the aim of this review was to explore the currently available data regarding this specific pathology. The complexity of the post-COVID-19-pandemic social context is also considered a reason to support the utility of such an exploration. The third factor that was taken into consideration was the existence of apparently contradictory data in the literature regarding the perspectives of pregnant women over their own body image (as previously mentioned), which indicates the need for finding specific risk factors for EDs in this population.

## 2. Objectives and methodology

The main objective of this rapid review was to identify relevant data about the risk factors, diagnostic criteria, epidemiology, pathophysiology, structured evaluation, and treatment of PN. The secondary objective was to determine if clinical recommendations could be made starting from the data retrieved in the literature.

Three major electronic databases (PubMed, Cochrane, and GoogleScholar) were searched using the paradigm “pregorexia” OR “eating disorders” AND “pregnancy” AND “risk factors” OR “diagnostic” OR “diagnosis” OR “prevalence” OR “incidence” OR “pathophysiology” OR “evaluation” OR “treatment” OR “therapy.” No inferior time limit was established, and the superior limit was January 2023. No restriction was applied to the language criterion. Both primary reports (case reports, case series, clinical trials) and secondary reports (systematic reviews, narrative reviews, meta-analyses) were allowed. Inclusion and exclusion criteria are presented in Table 1. Lists of references for each article were reviewed, and supplementary materials were investigated to see if they corresponded to the pre-determined objectives.

**Table 1:** Inclusion and exclusion criteria.

Operational criteria	Inclusion criteria	Exclusion criteria
Population	No age limits were established, but only pregnant women were allowed due to the specificity of the explored ED. The main diagnosis should be an ED detected during pregnancy, even if comorbid with other psychiatric and/or somatic disorders. No limitation of the diagnostic criteria was included. Clinical and general populations were allowed.	Absence of an ED during pregnancy as the main diagnosis.
Intervention	Any type of review method was allowed. Any type of study (clinical, preclinical, review) was admitted if it corresponded to the pre-defined objective of this review.	Studies with unspecified design, population, or applied statistical methods. Reviews that did not mention a search paradigm, an interval for papers collection, or the presence of search criteria that did not allow for a distinction between PN and other disturbed eating behaviors or attitudes.
Environment	Both in-patient and out-patient regimens.	Unspecified environment.
Primary and secondary variables	Evaluation of diagnostic criteria, epidemiology, pathophysiology, prevalence/incidence, risk factors, and treatment of PN and related EDs.	All research that was using unspecified or insufficiently described variables. Reviews without pre-defined quantifiable objectives.
Study design	Clinical trials, epidemiological studies, systematic reviews, narrative reviews, and meta-analyses. Longitudinal and transversal, retrospective or prospective studies. Animal model studies. Case reports and case series.	Studies with unspecified or insufficiently specified designs.
Language	Any language of publication was admitted if the *in-extenso* published paper was available.	

ED, eating disorder; PN, pregorexia nervosa.

## 3. Results

Data about the PN and other related EDs determined during pregnancy were grouped according to the main themes of interest, i.e., risk factors, diagnostic criteria, epidemiology, pathophysiology, structured evaluation, and treatment. It must be noted that data derived from good-quality studies on PN are missing, which can be explained by variability in the definitions of this entity. Data about EDs during pregnancy were included, even if they were not associated with the term „pregorexia”/„pregorexia nervosa,” in the presence of the core PN dimensions (onset during pregnancy, excessive dieting or physical exercising, and focus of attention on own body weight).

### 3.1. Risk factors

Women presenting body image distortions, a voluntary decrease in the calorie content of their meals, and excessive physical activity were enrolled in a gynecology center in Pakistan (N = 15 participants) and were investigated to detect the presence of EDs (Saleem et al., 2022). The tendency toward anorexia in this group was found in 40% of the cases, while the onset of specific EDs symptoms was observed in 60% of all cases (Saleem et al., 2022). The most commonly reported manifestations of PN were skipping meals, restriction of meals, control of calorie intake, strict dieting, taking fat-burn teas/laxatives/diuretics, eating alone, or eating too slow or too fast (Saleem et al., 2022). Almost 86% of these patients reported a good feeling related to increased diet control or exercise (Saleem et al., 2022).

The personal history of EDs should also be considered a vulnerability factor for the development of PN (Tuncer et al., 2020). Therefore, special attention should be directed to this subgroup during the monitoring of their pregnancy, with regular evaluation of eating habits, weight control, etc., as mentioned in the introductory chapter.

According to the National Institute for Health and Clinical Excellence (NICE), the risk of EDs should be taken into account when an unusually low or high BMI is observed; if rapid weight loss is detected; excessive dieting or restrictive eating practices exists; family members or carers report a change in eating behavior; social withdrawal when eating is involved; if concomitant mental health problems exist; a disproportionate concern about body weight or shape is identified; menstrual or other endocrine disturbances, unexplained gastrointestinal symptoms, malnutrition, misuse of laxatives, diet pills, emetics, or excessive exercise; abdominal pain associated with vomiting or dietary restrictions; electrolyte imbalance, hypoglycemia, atypical dental wear, problems managing a chronic illness that affects diet; participation in activities associated with a high risk of EDs (National Institute for Health and Care Excellence (NICE), 2020).

### 3.2. Positive and differential diagnosis

Several authors tried to define a clinical picture for PN, with obsessive thoughts related to weight gain as the core feature and with several associated signs, like the existence of any ED in personal history, discussing gravidity as if it were not present, an excessive focus on counting calorie, refusing to eat in the company of others, not take regular meals, etc. (Tuncer et al., 2020).

The patient’s interest in diet and exercise is disproportionate, and her preoccupation with the fetus’s health and acceptance of the change in body shape during pregnancy are less significant in the presence of a PN diagnosis (Tuncer et al., 2020). The patient’s focus of attention is their own body image and less on the pregnancy or the health of the newborn.

Lack of gaining weight during pregnancy could be the most critical single sign to detect a possible PN (Kubaszewska et al., 2012). To compare the evolution of this variable in women with EDs with a healthy population, it is advisable to use the weight gain milestones established by medical organisms or institutions, like IOM (American College of Obstetricians and Gynecologists, 2013).

**Figure 1:**
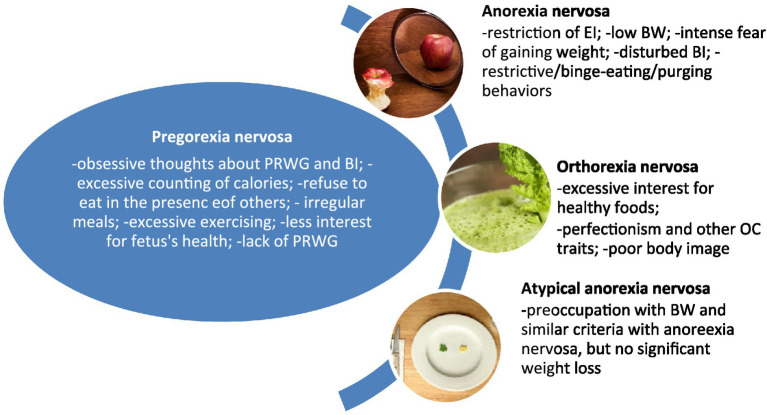
Specific and differential features of pregorexia nervosa. BI, body image; BW, body weight; EI, energy intake; OC, obsessive–compulsive; PRWG, pregnancy-related weight gain.

The differential diagnosis for PN includes mainly (1) anorexia nervosa and its atypical variant, and (2) orthorexia nervosa (Figure 1). Anorexia nervosa is the single most important differential diagnosis for PN. To differentiate these two disorders, it is necessary to explore the existence of the other EDs in the personal history. Anorexia nervosa is characterized by avoidance of energy intake leading to significant weight loss, intense fear of gaining weight or becoming fat, persistent behavior interfering with weight gain, in spite of significantly low weight, and disturbance of the own body weight or shape perception [American Psychiatric Association (APA), 2013]. A BMI value less or equal to 17 kg/m^2^ suggests the presence of anorexia nervosa [American Psychiatric Association (APA), 2013]. Atypical anorexia nervosa is defined by the same criteria, except for the body weight, which remains within or above the normal range, despite significant weight loss [American Psychiatric Association (APA), 2013]. PN shares with anorexia nervosa the focus of attention placed on body weight and excessive dieting/exercising, but PN is limited to a certain population, i.e., pregnant women, and associates specific manifestations, e.g., lack of interest in gravidity or other aspects related to the newborn.

Orthorexia nervosa is a controversial nosological entity, defined mainly by the obsessive interest in healthy foods, and disturbed eating habits leading to an unbalanced diet that negatively impacts health status and quality of life; emotional, cognitive, and/or social consequences have been described in patients with orthorexic habits; the symptoms are suggested to persist at least 6 months; low body weight may occur (McComb and Mills, 2019; Donini et al., 2022). Both PN and orthorexia nervosa are restrictive types of EDs, but PN does not necessarily involve a focus on healthy foods or extreme care in cooking meals.

### 3.3. Epidemiology

The determination of epidemiological parameters for EDs in pregnancy is negatively impacted by various factors, already exposed in the introductory chapter, varying from lack of validated instruments to demographic variables. However, according to a systematic review dedicated to EDs during pregnancy (*n* = 10 articles), no data about women who have symptoms of EDs with onset during pregnancy and sudden disappearance after delivery have been found (Janas-Kozik et al., 2021). Other sources reported prevalence values of 5% for pregorexia, determined during or after pregnancy, but the validity of this data is influenced by possible overlap with anorexia nervosa or orthorexia nervosa (Babicz-Zielinska et al., 2013).

### 3.4. Pathophysiology

**Figure 2:**
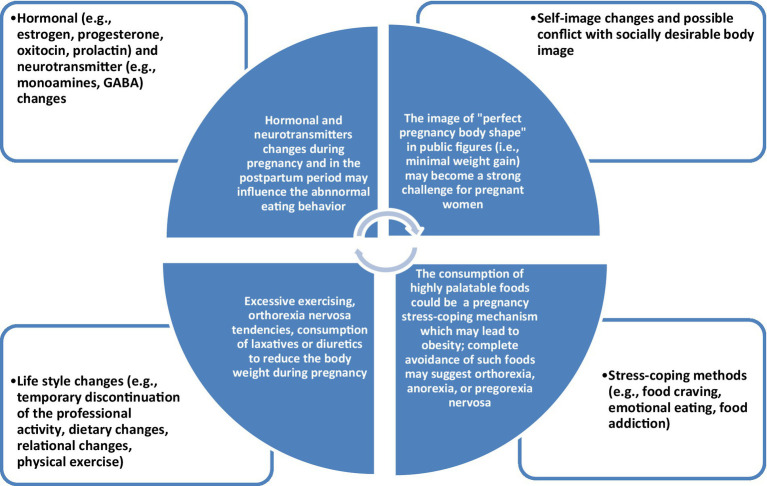
The complex interplay between biological, psychological, and social factors in pregorexia nervosa.

Hormonal and neurotransmitter changes, self-image distortions, social pressure for a perfect body image, lifestyle changes (professional, interpersonal, leisure activities, etc.), and coping strategies focused on pregnancy-related stress could contribute to the onset of PN (Figure 2). The data presented in this sub-chapter are mainly extrapolated from studies exploring EDs and pregnancy in general, not specifically PN. More preclinical and clinical studies are needed before firmly concluding the pathophysiology of PN.

#### 3.4.1. Hormonal and neurotransmitter changes

Adjustments in maternal hormone levels were explored in relation to mood variations during pregnancy and the postpartum period. Depression and anxiety were correlated with elevated cortisol levels, while depression was associated with acute changes in estradiol and progesterone levels (Zhou et al., 2009). Also, chronic maternal distress has a negative impact on the regulation of hormonal activity during pregnancy and elevates free *corticotropin-releasing hormone (CRH)* levels and CRH activity in the amygdala; even more, chronic maternal stress increased the incidence of anxiogenic and depressive-like behaviors in preclinical models (Weinstock, 2005). The reactivity of the *hypothalamic–pituitary–adrenal (HPA) axis* is reduced during pregnancy, as reflected in the results of human and translational studies; this decreased reactivity is considered a critical adaptation to protect the organism against gestational stress (Georgescu et al., 2021). *Prolactin* has been involved in food intake behavior and body weight in non-pregnant rats; in the pregnant state, rodents eat more and become insensitive to *leptin*, especially in the hypothalamic regions associated with leptin’s effects on food intake (Georgescu et al., 2021). Alterations in the *oxytocin* system during pregnancy have been explored in relation to a possible vulnerability toward developing postpartum depressive symptoms; levels of this hormone decreased from the 38th week of gestation to 2 days after delivery in participants with postpartum depressive symptoms, while its levels increased in the group without these symptoms (Jobst et al., 2016).

Hormones and neuroactive peptides have a significant impact on food reward circuits, and dysregulation in leptin or ghrelin functioning can alter eating behavior in anorexia nervosa and bulimia nervosa, according to data from basic research (Berner et al., 2018; Frank et al., 2019).

Pregnancy alters the regulation of *GABA, norepinephrine,* and *prolactin* as reflected in the cerebrospinal fluid (CSF) changes for these variables and could play a role in creating a vulnerability to anxiety and depression in this population (Altemus et al., 2004). Significant plasma decreases of *epinephrine, serotonin*, and *dopamine* have been reported in healthy, pregnant women in each trimester of pregnancy vs. non-pregnant women (Shetty and Pathak, 2002).

EDs have been associated with alterations in dopamine, acetylcholine, and opioid systems in reward-related brain areas that can translate into addictive or binge-eating behaviors (Avena and Bocarsly, 2012). Dopamine release is stimulated by binging hyper-palatable foods, while purging attenuates the release of acetylcholine, interfering with the signaling of satiety (Avena and Bocarsly, 2012). Restricted access to food enhances the reinforcing effects of dopamine in animal models, and alterations in mesolimbic dopamine and serotonin have been reported in preclinical paradigms of anorexia nervosa (Avena and Bocarsly, 2012).

Although no specific connections between hormonal and neurotransmitter dysfunctions, on one side, and PN, on the other, were found, it is expected that biological changes during pregnancy should elicit interest for further research in EDs. This would be of practical interest since finding adequate treatments involve the acknowledgment of the pathophysiological substrate for any disease.

#### 3.4.2. Social modulation of self-image

Satisfaction with the body image is dependent on multiple psychological factors, i.e., the difference between the current body image and the ideal body, or the emotional impact of body changes during different periods of life (Hawkins et al., 2004; de Sousa Silva et al., 2020). Repeated exposure to images of idealized bodies presented in the media creates supplementary stress for pregnant women, who are naturally gaining weight during their pregnancy (de Sousa Silva et al., 2020). The discrepancy between the ideals of beauty promoted by society and the real body of most people enhances the risk of dissatisfaction with own appearance, negative mood states, low self-esteem, and EDs (Myers and Crowther, 2009; de Sousa Silva et al., 2020).

Body image, body satisfaction, and dieting practices during pregnancy are modulated by social expectancies (Davies and Wardle, 1994). Although it is supposed that social pressures for slimness were less stringent in this period, studies conducted on this topic did not support such a hypothesis (Davies and Wardle, 1994). Dietary restrictions were lower in pregnant women and current attempts to lose weight were less frequently reported, but there was still no evidence that pregnancy was associated with more relaxed body image ideals (Davies and Wardle, 1994). According to a study (n_1_ = 76 pregnant women and n_2_ = 97 non-pregnant controls), pregnant women chose a figure of similar size to non-pregnant women as their ideal (Davies and Wardle, 1994).

The perception of the self-body image during pregnancy is extremely diverse and influenced by the strategies the women used to protect against the pressure of socially-constructed female beauty ideals (Hodgkinson et al., 2014). The women’s narratives about their bodies during pregnancy could represent the basis of self-dissatisfaction (Hodgkinson et al., 2014) and the activation of dysfunctional coping strategies. Health professionals are invited to explore the body image concerns in this population and to activate the necessary resources for accepting or decreasing body image dissatisfaction (Hodgkinson et al., 2014).

#### 3.4.3. Lifestyle changes

Diet and exercise are important both before and during pregnancy for the development of the child and for the mental and physical health of the mother (Koletzko et al., 2018). Recommendations for weight control during pregnancy, energy, and nutritional requirements and diet, supplementation with folic acid/folate, iron, and other macro-or micronutrients, physical activity, and avoiding alcohol and smoking have been presented by different healthcare organizations (Koletzko et al., 2018). According to the Healthy Start-Young Family Network, an appropriate weight gain in pregnancy is between 10 and 16 kg for women of normal weight, and in the case of overweight/obese women, a lower weight gain is desirable (Koletzko et al., 2018). In the case of underweight women, it is recommended that measures are taken to ensure sufficient weight gain during pregnancy (Koletzko et al., 2018). Pregnant women should increase their energy intake slightly (not more than 10%) and not until the last few months of pregnancy (Koletzko et al., 2018). Pregnant women should be moderately physically active for ≥30 min at least 5 days a week, preferably daily (Koletzko et al., 2018).

The interest of pregnant women in maintaining a healthy lifestyle was correlated with the degree they perceive themselves as healthy individuals (Morris et al., 2020). Women with health-disengaged attitudes were not interested in discussing their lifestyle, while those preoccupied with their own health did not feel the need for extra support (Morris et al., 2020).

Lifestyle changes during pregnancy were influenced significantly by preconceptions about lifestyle, the physiological demands of pregnancy, and the pressures of daily life (Walker et al., 2020). The lifestyle advice received from health professionals had a lower impact on maternal lifestyle behavior change than the socio-ecological environment (Walker et al., 2020). Adopting a pre-natal healthy lifestyle and losing weight is important for women and they are generally enthusiastic about programs that will help them achieve these goals, according to survey-based research (*N* = 126 respondents; Funk et al., 2015).

#### 3.4.4. Pregnancy-stress coping methods

Coping mechanisms in the context of pregnancy can influence the management of negative emotional, behavioral, cognitive, and physiological reactions to specific stressors (Guardino and Schetter, 2014). The type of coping strategies the individual activates and the functional impact of these strategies may represent a resource that protects both mother and children from prenatal stress exposure, but they may also represent a vulnerability. Avoidant coping behaviors and generally poor coping skills detected in pregnant women have been reported by a systematic review (*n* = 45 cross-sectional and longitudinal studies, *N* = 16,060 participants) as being associated with postpartum depression, preterm birth, and infant development (Guardino and Schetter, 2014). A functional coping strategy is taking steps to solve a problematic situation, while an example of a dysfunctional strategy is to avoid dealing with the stressor by developing different behavioral addictions or substance use disorders.

Positive coping strategies and higher perceived/received social support were associated with lower perceived stress, while evasive coping strategies were associated with higher levels of perceived stress (Goletzke et al., 2017). The need for psychoeducation and intervention aiming at improving social support and positive coping strategies, especially in multiparous women, have been suggested as strategies to reduce the risk for adverse pregnancy outcomes (Goletzke et al., 2017).

Maternal psychosocial stress, dietary behavior, and nutritional state are influencing one another during the pregnancy and this interplay has an impact on maternal metabolic health, fetal development, and offspring health outcomes (Lindsay et al., 2017).

Maladaptive dieting has been explored as a coping mechanism, based on escape-avoidance behaviors, and it has been reported more frequently in patients with past or current EDs (Ghaderi and Scott, 2000). Lower interest in seeking social support and successful problem-solving strategies has been reported in the same population, suggesting functional coping mechanisms are less used (Ghaderi and Scott, 2000).

### 3.5. Structured evaluation

Screening for EDs is recommended during pregnancy if the following risk factors are identified during the anamnesis: decreased BMI, intense worries related to body weight or shape, menstrual irregularities during adolescence, or even amenorrhea; different digestive symptoms, intrapsychic conflicts, clinical evidence of starvation, or recurrent, possible self-induced vomiting (Ward, 2008; Tuncer et al., 2020).

No specific scale for PN has been identified in the literature, but general instruments for EDs may be administered for screening purposes or for the evaluation of this disorder’s severity. While presenting all the instruments designed for the evaluation of ED symptomatology is outside the scope of this review, several of them could be used by clinicians and researchers interested in screening or monitoring the main features of PN. The *Eating Disorder Diagnostic Scale (EDDS)* is a brief self-administered instrument focused on the evaluation of anorexia nervosa, bulimia nervosa, and binge eating disorder, with good reliability and validity; an overall composite cut-off score of 16.5 distinguishes the clinical population from healthy controls (Krabbenborg et al., 2012). The *Eating Attitudes Test (EAT-40)* and its abbreviated, 26-item form (EAT-26) evaluates symptoms of bulimia, weight changes, body-image variables, and psychological symptoms; EAT-26 was also used for screening of binge eating disorder and eating disorders not otherwise specified in clinical nutrition unit attendees, and a cut-off of 11 was suggested (Garner et al., 1982; Orbitello et al., 2006). The *Eating Disorder Examination Questionnaire (EDE-Q)* is a brief, 36-item, self-administered instrument, created according to the Diagnostic and Statistical Manual of Mental Disorders (DSM-IV); acceptable internal consistency, test–retest reliability, and temporal stability have been demonstrated; a cut-off score of 4 on the global scale is considered clinically significant; there are four subscales focused on cognitive features of ED- „restraint,” „eating concern,” „shape concern,” and „weight concern” (Berg et al., 2012).

### 3.6. Treatment

Regarding the treatment of EDs during pregnancy, nutritional and psychosocial interventions are recommended (Tuncer et al., 2020). No specific therapeutic recommendations for PN have been detected in the literature. Case management conducted by a multi-disciplinary team is advisable, with the participation of obstetricians, mental health specialists, internists, and dietitians (Tuncer et al., 2020). Psychotherapy is considered the main intervention for pregnant women with associated EDs and mood disorders, as the pharmacological agents could have teratogenic effects or insufficient data to support their safety in this population (Petrescu et al., 2014; Vasiliu, 2019). Extrapolating data available in the case of other EDs, cognitive-behavioral therapy (CBT) seems to be the most supported by evidence intervention (Linardon, 2018).

The prevention of EDs in pregnant women should be considered an important objective of case management, as the consequences of neglecting such pathology may be severe in the long term. Particular attention should be given to women presenting risk factors, e.g., previous diagnoses of EDs, and psychoeducative interventions should help the patient distinguish between common complaints associated with pregnancy and possible signs of an ED (Kubaszewska et al., 2012). Re-orienting patients’ attention to the health of the fetus may help decrease the severity of worries related to their own body weight (Little and Lowkes, 2000; Kubaszewska et al., 2012).

## 4. Discussion

PN represents a still elusive nosological entity, and specific data available about its core dimensions are lacking, mainly due to its overlap with anorexia nervosa and orthorexia nervosa met during the pregnancy period. The interference of other EDs in this population cannot be excluded, either. To further complicate the investigation of PN, no clinical instruments have been validated for this specific disorder, although items from the existing scales and questionnaires can be used for screening the vulnerable population. Taking into account the main vulnerability factors, out of which lack of expected weight gain is the most important alarm signal, it is extremely important to define the most exposed individuals to PN. The complex interplay of biological (i.e., hormonal and neurotransmitter changes), social (e.g., idealized body image, mass media campaigns for promoting „perfect pregnancy body shape”), and psychological (e.g., coping mechanisms and self-image) factors are presumed to combine with pre-pregnancy vulnerability (e.g., specific EDs or isolated symptoms of EDs) in generating new EDs, like PN, or the reactivation of pre-existing EDs.

The treatment is based on psychosocial interventions, mainly CBT, but there are no well-defined strategies. Prevention is also important, and screening for PN in vulnerable populations may be helpful for initiating early interventions. Psychoeducation about lifestyle changes, dieting, and physical exercise in pregnant women may be tempted as a preventive measure for EDs.

## 5. Conclusion

Exploring the clinical and epidemiological aspects of PN is considered essential for the practice-health promotion in the vulnerable population of pregnant women. Early detection through screening by GPs and obstetricians during the monitoring of pregnancy could be helpful in improving the quality of life and mental health of these patients. Referring patients with symptoms of PN to psychologists, psychotherapists and/or psychiatrists may improve the outcome of the pregnancy and the normal development of the newborns.

The importance of EDs during the actual, post-COVID-19-pandemic context, has been extensively explored (Zipfel et al., 2022) and it was found that the burden of this pathology has increased, signaling the need for raising awareness in the general population about EDs. A particular detrimental effect of the pandemic on people with a high risk of EDs onset is reflected in the increased incidence of disturbed eating behaviors or attitudes with up to 15.3% in 2020 vs. the previous year (Taquet et al., 2021). Therefore, an improvement in the early detection of EDs in primary care settings, a reduction in the duration to access specialized treatment, an increase in the efficacy of routine therapeutical management, an optimization of inpatient care, and finding new rehabilitation approaches for patients who do not respond to standard treatment are needed (Treasure et al., 2020). Exploring the risk factors and optimal therapeutical approaches for patients with PN should be integrated into this global approach.

Regarding the limitations of the current research, it should be mentioned that it is a rapid review, therefore it is possible that not all relevant materials have been retrieved. Also, no quality assessment of the reviewed papers was included, since the relevance of such an approach if only one author is involved could be very limited. The strength of the review consists mainly in the importance of its topic, and the fact that information about the core categories used to describe any medical condition has been selected, i.e., pathophysiology, epidemiology, clinical diagnostic, psychometric measurements, and treatment. Future research dedicated to all these aspects of PN is granted, based on (1) the need to preserve a good quality of life and mental health in pregnant women; (2) the importance of EDs in the post-COVID-19-pandemic period; (3) the clinical relevance of differentiating PN from other EDs, in order to find the most adequate therapeutic approaches for these patients. The most important topics to be addressed by forthcoming research are (1) validation of diagnostic criteria for PN, based on clinical and non-clinical samples; (2) screening tools for vulnerable populations, especially pregnant women with EDs history; (3) psychotherapeutic approaches targeting PN symptoms; (4) long-term follow-up studies exploring the risk of PN recurrence during the following pregnancies, and the risk of the onset of other EDs in patients with a history of PN; (5) the impact of PN over the physical health of the fetus and, on the long-term, over the children’s physical and mental health.

## Author contributions

The author confirms being the sole contributor of this work and has approved it for publication.

## Conflict of interest

The author declares that the research was conducted without any commercial or financial relationships that could be construed as a potential conflict of interest.

## Publisher’s note

All claims expressed in this article are solely those of the authors and do not necessarily represent those of their affiliated organizations, or those of the publisher, the editors and the reviewers. Any product that may be evaluated in this article, or claim that may be made by its manufacturer, is not guaranteed or endorsed by the publisher.
